# Call to action for genetic counseling research in hereditary cancer: Considerations from the evidence‐based guidelines development process

**DOI:** 10.1002/jgc4.70026

**Published:** 2025-04-30

**Authors:** Brooke Levin, Erin Salo‐Mullen, Julie O. Culver, Raluca N. Kurz, Pamela Brock, Rochelle Demsky, Stacy Lloyd, Ghecemy Lopez, Phuong L. Mai, Kami Wolfe Schneider

**Affiliations:** ^1^ MD Anderson Cancer Center at Cooper Cooper University Hospital Camden New Jersey USA; ^2^ Memorial Sloan Kettering Cancer Center New York New York USA; ^3^ USC Norris Comprehensive Cancer Center University of Southern California Los Angeles California USA; ^4^ College of Science and Health Charles R. Drew University of Medicine and Science Los Angeles California USA; ^5^ Department of Internal Medicine The Ohio State University Columbus Ohio USA; ^6^ LifeLabs Genetics Toronto Ontario Canada; ^7^ VHL Alliance Boston Massachusetts USA; ^8^ Celebrate Life Cancer Ministry Hawthorne California USA; ^9^ Center for Clinical Genomics and Genetics University of Pittsburgh Pittsburgh Pennsylvania USA; ^10^ Department of Pediatrics University of Colorado Anschutz Medical Campus Aurora Colorado USA

**Keywords:** evidence‐based guidelines, genetic counseling, genetic testing, hereditary cancer, risk assessment

## Abstract

The National Society of Genetic Counselors (NSGC) planned to develop an evidence‐based guideline on the outcomes of genetic counseling for individuals at risk for hereditary cancer. The practice guideline workgroup used Grading of Recommendations Assessment, Development and Evaluation (GRADE) methodology including ranking the importance of outcomes of genetic counseling for individuals at risk for hereditary cancer. However, due to evidence gaps in the literature, particularly the limited availability of high quality and well‐designed studies for many important outcomes of genetic counseling, the NSGC identified a need for additional research prior to guideline development. Herein, we describe a “call to action” for future research, particularly for health services‐related outcomes of genetic counseling in diverse populations. Identified research priorities include conducting high‐quality studies that separate the outcomes of genetic counseling from genetic testing, assessing outcomes associated with pre‐ and/or post‐test genetic counseling, measuring patient‐reported and health system‐reported outcomes, comparing genetic counseling by certified genetic counselors versus non‐genetics‐trained providers, differentiating need in various hereditary cancer indications, and identifying barriers to genetic counseling in historically excluded patient communities.

**Purpose**

- To describe a multidisciplinary workgroup's approach to applying the GRADE methodology to develop a guideline assessing the outcomes of genetic counseling for individuals at risk for hereditary cancer.
- To highlight the most important outcomes of cancer genetic counseling.
- To identify gaps in evidence in genetic counseling research and to identify future research priorities.

## 1 INTRODUCTION

Hereditary cancer predisposition is increasingly recognized as contributing to cancer diagnoses (Esplin et al., 2022). Existing professional society consensus recommendations and payors' hereditary cancer genetic testing policies outline indications for consideration of germline genetic testing, including family history of cancer, age at diagnosis, type of cancer, and/or pathological or molecular characteristics of the tumor (Druker et al., 2017; Manahan et al., 2019; NCCN, 2024a, 2024b). Identification of hereditary cancer predisposition may have medical and psychosocial implications that genetic counseling can address.

Genetic counseling is defined as “the process of helping people understand and adapt to the medical, psychological and familial implications of genetic contributions to disease” and includes interpretation, education, and counseling (Resta et al., 2006). Genetic counseling encompasses not only risk assessment and facilitating genetic testing but also lifelong support for individuals and their families living with genetic risk or conditions. In addition to outlining criteria for genetic testing, professional society and consensus guidelines often recommend risk assessment and genetic counseling for individuals suspected to carry a germline variant in a gene related to cancer predisposition (Bedrosian et al., 2024; Berliner et al., 2021; Hampel et al., 2015; NCCN, 2024a, 2024b; Owens et al., 2019). Further, cancer center accrediting bodies include requirements for provision of genetic counseling and risk assessment (American College of Surgeons, Commission on Cancer Standards, 2020).

A systematic review of international guidelines for individuals with *BRCA*‐related cancer predisposition, however, notes that the timing and format of genetic counseling are not always well‐described among guidelines (Forbes et al., 2019). Much of the evidence for genetic counseling in hereditary cancer is for individuals at risk for hereditary breast and ovarian cancer, and the populations studied have lacked diversity, primarily focusing on cisgender women of White, European ancestry. While traditional models of genetic counseling include face‐to‐face pre‐ and post‐test counseling sessions, the implementation of alternative service delivery methods (e.g., audio‐only sessions, patient education videos, printed materials, or chatbots) is of interest in clinical and research settings to enhance—or in some cases replace—the “gold standard” pre‐ and post‐test genetic counseling paradigm (Buchanan et al., 2016; Kaphingst et al., 2021; McCuaig et al., 2018; Mittendorf et al., 2021; Pozzar & Seven, 2023; Rayes et al., 2019).

Existing systematic reviews regarding outcomes of genetic counseling in various settings, including hereditary cancer, prenatal, pediatric, and general adult genetics, have primarily addressed patient‐reported outcomes such as psychological well‐being, knowledge, perceived risk, patient satisfaction, and genetic testing decisions (Athens et al., 2017; Braithwaite et al., 2004; Cirino et al., 2022; Danylchuk et al., 2021; Hilgart et al., 2012; Madlensky et al., 2017; Meilleur & Littleton‐Kearney, 2009; USPSTF, 2019). It is noted that the measures used for patient‐reported outcomes in genetic counseling were initially validated in primarily White, European patient populations, though cross‐cultural validation studies are also underway (Kasparian et al., 2007; McAllister et al., 2011; Segundo‐Ribeiro et al., 2020; Yang et al., 2024). As of 2024, only one US‐based hereditary cancer guideline includes a systematic evidence review to outline a justification for genetic counseling, separately from the justification for genetic testing for hereditary breast and ovarian cancer (USPSTF, 2019).

To develop evidence‐based guidelines for cancer genetic counseling, the NSGC practice guidelines committee organized a systematic review of the literature and guidelines panel to evaluate the review and make recommendations. Culver et al. (2024) conducted the systematic evidence review (SER) and meta‐analysis to evaluate the impact of genetic counseling on outcomes for individuals undergoing cancer genetic risk assessment. Culver et al. (2024) identified data to support that pre‐test genetic counseling significantly decreased worry, increased knowledge, and decreased perceived risk, but the certainty of the evidence was low according to the Grading of Recommendations Assessment, Development and Evaluation (GRADE) methodology (Balshem et al., 2011). Also, very little data were available regarding the effect of genetic counseling on health services‐related outcomes such as ordering the correct test, increasing the accuracy of risk assessment, reducing inappropriate services, improving cancer prevention behavior, or increasing cascade testing (Culver et al., 2024). Subsequently, the workgroup tasked with creating guidelines utilizing GRADE Evidence to Recommendation methodology determined that data were insufficient to formulate evidence‐based guidelines for the role of genetic counseling in hereditary cancer at this time (Guyatt et al., 2008). Instead, the following summary reviews the process undertaken by this workgroup, the issues and insights learned, and highlights the need for and importance of future research that would address gaps in the current literature.

Of note, NSGC‐endorsed systematic evidence reviews and guideline development projects in other domains of genetic counseling were successfully undertaken within similar timelines. Three evidence‐based guidelines have been published as of 2024, including recommendations regarding telehealth genetic counseling, genetic testing for unexplained epilepsies, and expanded carrier screening (Green et al., 2023; Sagaser et al., 2023; Smith et al., 2023). These guidelines address the implementation of telehealth versus in‐person genetic counseling, outcomes of genetic testing for individuals with epilepsy, and expanded carrier screening versus ethnicity/race‐based carrier screening, respectively. These guidelines examine modalities of genetic counseling service delivery and outcomes of genetic testing. The epilepsy guideline does include a strong recommendation for genetic testing in the context of formal pre‐ and post‐test genetic counseling by a qualified healthcare provider; however, the authors acknowledge that the benefits and risks have not been systematically studied (Smith et al., 2023). In contrast, the goal of the hereditary cancer SER and guideline was specifically to assess evidence‐based outcomes of genetic counseling separate from genetic testing for individuals at risk for hereditary cancer, with this evidence largely being limited to support a strong recommendation.

## 2 METHODS

The National Society of Genetic Counselors (NSGC) had a goal to develop an evidence‐based guideline on the role of genetic counseling for individuals at risk for hereditary cancer. In 2016, the NSGC Practice Guideline Committee (NSGC‐PGC) solicited volunteers for systematic evidence review (SER) and practice guideline (PG) workgroups to focus on hereditary cancer genetic counseling. The goal of this effort was to create a guideline following the Institute of Medicine (IOM) standards for developing clinical practice guidelines (IOM, 2011). Prior to beginning the SER, the two workgroups met together to develop the population (P), intervention (I), comparator (C), and outcomes (O) and study design (S) (PICOS) process and outcome prioritization in 2017–2018.

Details of the PICOS development process followed the GRADE framework (Guyatt et al., 2011) and is detailed in Culver et al. (2024). Briefly, the PICOS definition included (P) individuals or families undergoing genetic counseling for hereditary cancer risk, (I) genetic counseling by a health care provider with specialized training in cancer genetics (e.g., genetic counselor, MD geneticist, advanced practice genetics nurse, or other medical professional with specialized, high‐level training in cancer genetics) and (C) compared (1) no intervention, (2) education by a non‐genetics trained provider (e.g., gynecologist, surgeon, oncologist), and/or (3) other intervention (e.g., printed education materials).

The SER measured the impact of genetic counseling for individuals undergoing hereditary cancer risk assessment. The relevant outcomes to evaluate for this measurement were selected to include not only patient‐related (O) outcomes (positive effects including improvements in health behavior, health outcomes, intent to pursue genetic testing or receipt of genetic testing, knowledge, perceived risk, psychosocial well‐being, satisfaction, and shared decision‐making, as well as potentially negative impacts including time spent/patient burden) but also the health services‐related outcomes (positive impacts including correct genetic test ordered, correct interpretation of the genetic test result, correct assessment of cancer risk, cost‐effectiveness, care coordination, family outcomes, and reduction in unnecessary services, as well as potentially negative impacts including delay in care). These include outcomes that were assessed in previous systematic reviews (Braithwaite et al., 2004; Hilgart et al., 2012; Meilleur & Littleton‐Kearney, 2009; Owens et al., 2019) as well as additional health services‐related outcomes. The accepted study design (S) included randomized controlled trials (RCT) and only considered observational studies if RCTs were unavailable. Due to the acceptable study designs, many articles related to genetic counseling were excluded.

The PG workgroup was tasked with creating guidelines on genetic counseling for individuals at risk for hereditary cancer utilizing the GRADE Evidence to Recommendation methodology. The PG workgroup initially consisted of seven genetic counselors with a range of clinical experience, practice areas, and geographic locations (six US‐based and one Canada‐based) who participated in the original PICOS development process and outcome prioritization with the SER workgroup. In 2019, one PG workgroup genetic counselor member withdrew and was replaced by a medical oncologist. Upon completion of the SER and subsequent handoff to the PG workgroup, two patients, one each representing a common and a more rare hereditary cancer syndrome, joined the workgroup prior to the commencement of guideline‐related activities in January 2021. The workgroup also included two patient advisors who provided feedback and additional patient perspectives but were non‐voting members. The addition of these non‐provider group members is crucial for the Evidence to Decision framework according to GRADE methodology (Schünemann et al., 2014). All voting members and their potential conflicts of interest were reviewed by the NSGC‐PGC prior to appointment, and the group was guided through the process with contracted methodologist support.

Given the passage of time since the original outcome prioritization and the changes to the workgroup membership, re‐prioritization of outcomes was pursued. These discussions included workgroup members only and did not include the enrollment of human subjects; therefore, this was not submitted for review by an institutional review board/human investigations committee/ethics committee. Prior to outcome ranking, a group discussion was held to review the outcomes initially ranked by the SER and PG author group members in 2018. At this time, there was the opportunity to suggest additional outcomes not previously included. Then, each member was asked to rate the importance of previously identified outcomes anonymously via Google Forms. Outcomes were ranked on a nine‐point scale defined by GRADE terminology, where 1–3 were considered “not important” 4–6 were “important” and 7–9 were “critical” for making a recommendation for genetic counseling for individuals at risk for hereditary cancer. Individual responses were aggregated and presented in a bar graph visual during group discussions when final rankings were assigned. The workgroup then held twice‐per‐month meetings from May 2021 to August 2021 to review the poll results and develop consensus for discussion and prioritization of both patient‐related and health services outcomes of genetic counseling for individuals at risk for hereditary cancer. Each outcome was given a suggested starting rank based on poll results. During the discussions, both voting and non‐voting workgroup members provided input on ranking—beginning with the patients followed by the genetic counselors and physician. The group co‐leaders then led discussion and suggested a final ranking. A quorum (80%) of workgroup members then agreed to the final ranking. The outcomes of the PICOS included in the SER and PGC workgroup discussions are outlined below.

Of note, the workgroup originally considered a GRADE‐ADOLOPMENT approach to guideline development given the presence of existing guideline statements and consensus recommendations which reference genetic counseling for individuals undergoing risk assessment for hereditary cancer. Following the approach outlined by Schünemann et al. (2017), the workgroup identified existing guidelines via an ECRI guidelines trust literature search and via a citation scan of the retrieved articles (https://guidelines.ecri.org). The group ultimately deemed that the ADOLOPMENT approach would not sufficiently address the aim of the current guideline given the lack of evidence addressing the genetic counseling outcomes the workgroups had ranked. Following the completion of outcomes ranking and determining that the ADOLOPMENT approach was insufficient, the members began a de novo GRADE Evidence to Decision framework for the creation of a guideline in September 2021.

## 3 OUTCOMES RANKING

Initially, the SER and PG workgroups ranked 18 outcomes of genetic counseling for individuals at risk for hereditary cancer following a similar process as noted in the Methods section. The current discussions allowed for flexibility to add or remove outcomes, as well as further define the outcome. Two outcomes included in the initial SER—shared decision‐making and care coordination—were removed. Care coordination was removed from the final SER analysis because the National Quality Foundation (NQF) changed the metric—it is now related to advanced care plans for patients 65+ years. Shared decision‐making was removed based on suggestion from consultation with GRADE methodologists, as shared decision‐making should be considered an underlying principle of patient care. Through the re‐ranking process with addition of patient perspectives, two additional outcomes were added, cascade testing and patient empowerment. Justification included that cascade testing could be considered separate from “other family outcomes” and that patient empowerment was a possible outcome of genetic counseling not captured in the psychosocial well‐being. Other notable differences between the two ranking processes following the addition of patient perspectives include elevation in the importance of psychosocial outcomes, knowledge, and risk perception after genetic counseling to “critical” whereas they were previously considered “important.” Reduction of inappropriate services was downgraded as “important” from its initial rank of “critical.” Patients highlighted the importance of patient autonomy while valuing the important role of genetic counselors in providing psychosocial counseling, education, and decision support. Health behavior, health outcomes, correct test ordered, and reduction in inappropriate services ranking discussions were also influenced by patient perspectives in that these were in general considered more patient‐specific (i.e., relevant to patient autonomy) and/or not a direct outcome of genetic counseling (i.e., health outcomes not considered within the scope of a genetic counselor's practice). A summary of the ranking, definitions, and main discussion points of each outcome are outlined in Tables 1A, 1B, and 1C. Within each table, outcomes are listed in alphabetical order.

**TABLE 1a:** Critical outcomes of genetic counseling (Rating scale 7–9).

Outcome (*n* = 11)	Discussion points
Appropriate management recommended	Medical management recommended was appropriate given the patient's assessed risk. It is noted that GC professionals provide the education around the available medical management options, assuring that they are appropriate and individualized for the patient. Non‐GC healthcare providers may also provide genetic counseling and make medical recommendations.
Correct test ordered	The group acknowledges that there may be more than one genetic test that is a consideration for a particular patient. Therefore, this outcome may also be defined as “appropriateness” of genetic test ordered.
Family outcomes: cascade testing	Cascade testing was separated from “other family outcomes.” Group discussion included GC ability to provide support for patients to share information with family members, this is seen as an important role of profession. However, follow‐up whether family members are informed and/or elect to proceed with testing may not be feasible given real‐world HIPAA and confidentiality issues.
Health behavior	Patient adherence to medical management recommendations made by the provider (e.g., mammography or colonoscopy) based on hereditary cancer risk. Patients' perspective: Health behavior outcomes should also consider patient autonomy, so genetic counseling may not always have its intended outcome. Consider incorporation of general population lifestyle modifications (e.g., American Cancer Society, cancer risk, and prevention).
Health outcomes	Reduction of cancer risks and morbidity, and longer lifespan for patient/population at risk for hereditary cancer. This includes both patients with and without a diagnosis of a cancer/tumor. Patients' perspective: Health outcomes may not be seen as the primary role of the counselor (e.g., role of genetic counselor is not to “cure” cancer).
Patient empowerment	“A process through which people gain greater control over decisions and actions affecting their health” (World Health Organization, 2021). The impact of genetic counseling on patient empowerment in the cardiac genetics setting has been examined by Murray et al. (2022).
Patient knowledge	Patient understanding of genetics, genetic information, inheritance, implications of a pathogenic variant. From the patients' perspectives, genetic counseling can help to provide a broad overview of a hereditary cancer syndrome, compared to the individual focus of each medical specialist, therefore providing patients with comprehensive knowledge is very important.
Provider ability to interpret test correctly	Includes interpretation of positive, negative, and uncertain genetic test results.
Psychosocial outcomes	Patient's psychological well‐being including anxiety, distress, depression, quality of life, intrusive thoughts, self‐esteem, decisional regret. It is noted that there is a possible overlap though important professional distinction between genetic counseling scope of practice and professional counseling therapy. From the patients' perspective in this group, “counseling” is a core piece in the genetic counseling process. It is noted that this aspect may not be considered critical for all patients and is dependent on the indication, testing process, etc.
Risk perception	Cancer risk or genetic risk perceived by patient is aligned with the patient's calculated risk. Correspondence to patient knowledge. Risk perception may be seen as integral to the GC process. However, perception of risk may not be as important as a patient's actions/health behavior. Correspondence to psychosocial outcomes (e.g., if patient believes that risk is “high” when in fact it is likely average, psychosocial counseling can help lead to more appropriate risk perception).
Shared decision‐making^a^	The extent to which a patient is involved in the decision‐making process. There are currently NQF instruments designed to measure this outcome, which would be considered separately from psychosocial outcomes. A specific hereditary cancer genetic testing tool to measure patient perceptions of shared decision‐making for genetic testing has recently been evaluated for feasibility, acceptability, reliability, and validity (Gore Moses et al., 2023).

^a^ Shared decision‐making was later removed in December 2021 based on conversations with consultant methodologists, Emma Bohn and Yngve Falck‐Ytter (Evidence Foundation).

**TABLE 1b:** Important outcomes of genetic counseling (Rating scale 4–6).

Outcome (*n* = 5)	Discussion points
Cost‐effectiveness	Cost‐effectiveness is a separate variable in the Evidence to Decision GRADE framework (Xie et al., 2023). Aspects of cost‐effectiveness were considered, with discussions that these aspects do not all fit the model for a traditional cost‐effectiveness analysis: ⚬ Costs for the individual/patient population versus for the healthcare system. ⚬ Genetic counseling service providers (whether physician versus advanced practice provider vs. genetic counselor) could factor into the associated costs of genetic counseling for the individual or payor. ⚬ Provider effectiveness in ordering correct testing could impact costs for the individual, payor, and / or health system. Genetic counselors can provide patient education regarding access to free familial variant testing, financial assistance for genetic testing, and other care coordination roles.
Delay in care	This outcome definition could also include “timeliness” of care to indicate a continuum rather than only a negative outcome. Group discussion included notifications of several points in care at which timeliness could be a consideration: ⚬ Delay in medical intervention / treatment for patients while waiting for genetic counseling / genetic testing and subsequent post‐test counseling (e.g., delaying breast surgery). ⚬ Delay in genetic testing for patients while awaiting appointment for pre‐test genetic counseling. Consider timeliness related to referral and scheduling with potential implications of alternative service delivery models.
Other family outcomes	Non‐cascade testing outcomes could include patient communication of genetic test results with at‐risk relatives, genetic testing of living, affected relatives, as well as psychosocial and health outcomes of family members. The scope of genetic counseling was considered in ranking this outcome as “important” rather than “critical.” Genetic counselors may have limited direct impact on most family member outcomes. Concerns regarding measurability of this outcome and quality of evidence also taken into consideration in ranking of this outcome.
Patient satisfaction	Patient's perceived satisfaction with genetic counseling. Typical post‐visit surveys may aim to assess overall consumer sentiment with a healthcare system or provider, versus other components of patient satisfaction with the process of genetic counseling. From the patients' perspective, when answering surveys regarding satisfaction, patients may consider the overall “service” provided rather than the specific provider or intervention.
Reduction in inappropriate services	Alternate terminology: “reduction of unnecessary services” Consider population included in PICO. ⚬ Patient/population: Reducing unnecessary genetic testing, procedures, cancer screening, and / or other healthcare services for the individual. ⚬ Health systems: Utilization review and potential barriers to testing (role of genetic counselor in utilization management) for the healthcare system. This outcome was considered “less important” from the patient/population perspective.

**TABLE 1c:** Less important outcomes of genetic counseling (Rating scale 1–3).

Outcome (*n* = 2)	Discussion points
Time spent	A potential barrier to services, including time spent in appointment, transportation, travel, wait times, and overall convenience of care. Time spent to have genetic counseling (burden from the patient perspective).
Uptake/intent to pursue genetic testing	Whether the patient elected to have genetic testing or intended to have testing.

## 4 EVIDENCE TO DECISION PROCESS

The NSGC‐PGC provided external, contracted expert methodology support for consultation to assist with the challenge of providing a recommendation utilizing the GRADE Evidence to Decision framework. Though the SER conducted by Culver et al. (2024) did identify a positive impact of genetic counseling on the patient‐reported psychosocial outcomes, the quality of the evidence rated using GRADE methodology was low or very low. Further, there were equivocal findings regarding many of the critically ranked outcomes. While the evidence does not suggest harm from genetic counseling, considering the differences between the variety of cancer types and genetic conditions for which genetic counseling could be provided, evidence separating the benefits of genetic counseling from genetic testing is limited. Therefore, providing an evidence‐based recommendation regarding the intervention of genetic counseling for individuals at risk for hereditary cancer based on low or very low‐quality evidence by systematic review was deemed to be premature. To reduce the perceived bias of a guideline on the intervention of genetic counseling based on limited evidence, with the endorsement of the NSGC‐PGC, the workgroup decided to defer the completion of a formal guideline and rather shift focus to highlighting the issues, challenges, and opportunities in this guideline development process with a call for considerations for different approaches to establishing guidelines and further research to evaluate the impact of cancer genetic counseling. Research designs were outlined by one PG group member (RK) and reviewed by all members on subsequent workgroup calls. Table 2 highlights the study designs proposed to address the questions of the benefits, risks, and limitations of the process of genetic counseling for individuals at risk for hereditary cancer syndromes.

**TABLE 2:** Examples of research ideas for well‐designed studies to measure different outcomes of genetic counseling are provided.

Research Goal	Evaluate impact of genetic counseling separate from impact of genetic testing	Assess health system reported outcomes	Compare GC performed by CGC vs. GC by non‐CGCs	Identify barriers to GC in historically excluded patient communities
2 Methodology / Design, examples:	Randomized, observational, and / or cross‐sectional studies with various timing of intervention in process of GC. Important to consider definition of GC and participant access to information and support: GT only versus GT and GC (pre‐ AND post‐ test); This should separate the effect of GC from GT. GT with pre‐test GC versus no pre‐test GC; all participants undergo GT and all receive post‐test GC. GT with post‐test GC versus no post‐test GC; all participants undergo GT and all receive pre‐test GC.	Chart review electronic medical record study or payor claims study. Assess outcomes for patients who underwent GT with / without GC (pre‐test and / or post‐test)	Randomized, observational, and / or cross‐sectional studies with variance in provider type. Consider three comparators: CGC Non‐CGCs but trained in cancer risk assessment Non‐ CGCs not trained in cancer risk assessment	Exploratory, qualitative research (e.g., focus groups) Observational study measuring barriers Interventional study to implement and assess timing, method, delivery of GC to reduce barriers and increase access
3 Measurable outcomes				
(a) Patient‐related: Psychosocial well‐being Knowledge Risk perception Family outcomes/cascade testing Empowerment Satisfaction Time spent/patient burden Update/intent to pursue genetic testing	X		X	X
(b) Health‐related: Health outcomes Health behavior Delay in care	X	X	X	X
(c) Provider‐related: Provider ability to interpret test correctly Correct test ordered			X	
(d) Care system‐related: Reduction in inappropriate services Cost‐effectiveness		X	X	

*Note*: Row 1 contains a general research aim. Row 2 contains study design examples. Row 3 (a–d) contains sub‐categories of outcome types to be assessed under each aim/study design.

Abbreviations: CGC, certified genetic counselor; GC, cancer genetic counseling; GT, cancer genetic testing; Non‐CGC, health care provider with specialized training in cancer genetics (e.g., MD geneticist, advanced practice genetics nurse, or other medical professional with specialized, high‐level training in cancer genetics); RCT, randomized controlled trial.

## 5 DISCUSSION

Though the process outlined above did not result in the establishment of an evidence‐based guideline, lessons learned can guide future research in the outcomes of genetic counseling for individuals at risk for hereditary cancer. The issues, insights, and recommendations for future research are outlined below.

### 5.1 Evaluating outcomes of genetic counseling independently from genetic testing

The SER focused on the need for evidence to specifically support practice‐based guidelines for hereditary cancer genetic counseling, separately from genetic testing. With these definitions in place, it was important that the SER team limit the review to reports from which the effect of genetic counseling was measurable and distinguished from the effect of genetic testing. During the SER team's study identification process, 1042 studies were excluded based on the effect of genetic counseling not being measurable or unable to be distinguished from the effect of genetic testing, while only 25 studies were, in the end, included in the report (Culver et al., 2024). This imbalance in the number of relevant publications indicates the need for future research studies that specifically evaluate the effects of hereditary cancer genetic counseling.

### 5.2 Patient and provider involvement

The incorporation of patient advocates and both physician and genetic counselor provider representation in the guideline workgroup was impactful to the process. Inclusion of diverse perspectives provides the priorities for groups impacted by the guideline; in this case, the patients indicated the critical nature of psychosocial and knowledge outcomes of genetic counseling for hereditary cancer and a de‐emphasis on outcomes such as reducing mortality and cancer screening. Future practice guideline workgroups may wish to incorporate viewpoints of representatives from both provider and patient communities, including those from diverse backgrounds.

### 5.3 Genetic counseling currently accepted as “standard of care”

Existing hereditary cancer‐related guidelines include a recommendation for genetic counseling (Berliner et al., 2021; Hampel et al., 2015; NCCN, 2024a, 2024b; Owens et al., 2019). Further, certain payors also require pre‐ and post‐test genetic counseling for all hereditary cancer syndrome genetic tests (e.g., Cigna, Medical Policy Coverage Number 0518, July 15, 2022). These recommendations are largely based on expert opinion and historical precedent and could present possible challenges in justifying future studies with comparator groups receiving “no genetic counseling” which would allow for assessing the outcomes of pre‐ or post‐test traditional genetic counseling separate from genetic testing. Despite these precedents, research has demonstrated that a majority of genetic testing is performed outside of a genetics clinic and that 50% or less of patients undergoing hereditary cancer genetic testing receive pre‐ or post‐test genetic counseling from certified genetic counselor or other genetics professional (Armstrong et al., 2015; Bajguz et al., 2022; Cragun et al., 2015; Kurian et al., 2017). Current research studies evaluating alternative service delivery models may help to address the relative risks, benefits, and limitations of more streamlined practice to standard pre‐ and post‐testing genetic counseling, which does in fact already occur in many clinical settings (Kaphingst et al., 2021; Mittendorf et al., 2021; Rayes et al., 2019). Similarly, additional well‐designed studies will help provide measurement and improve understanding of how this standard practice impacts patient and health service‐related outcomes.

### 5.4 Evidence‐based guideline methodologies

An important process of GRADE methodology includes grading of the evidence base to form a recommendation. The hierarchy of evidence that places (RCTs) at the top was developed for clinical trials that test the efficacy and safety of drugs and devices. RCTs with genetic counseling, however, may not be feasible, ethical, or practical if a study arm omits access to all forms of genetic counseling. Genetic counseling is a complex intervention. For meaningful RCTs to evaluate genetic counseling as an intervention, studies should consider the provider type, delivery methods, content, and patient characteristics, among other variables, while not limiting access to appropriate information and support.

Due to challenges with the complexity and standard acceptance of genetic counseling, in addition to RCTs, the effect of genetic counseling can be studied with well‐designed observational studies (such as cohort and case–control) Studies should specifically address various delivery models, the timing of genetic counseling within a care pathway, provider type, diverse and specific populations, and more. Ideally, studies should strive for the highest‐quality design that is feasible and not restrictive to high‐quality care.

## 6 RECOMMENDATIONS

### 6.1 Diversity in genetic counseling research

As the group considered outcome prioritization, addressing the needs of diverse populations was considered a critical outcome; however, the group ultimately determined that diversity, equity, and inclusion in genetic counseling for hereditary cancer need to be embedded in every outcome and not as a stand‐alone outcome. An existing systematic review showed that research on genetic counseling in diverse populations, while present, lacks size and consistent structure for examining differences in genetic counseling participation, knowledge and awareness, motivators, barriers, perceptions, and outcomes among White and non‐White groups (Southwick et al., 2020). It has been demonstrated that historically excluded populations are less likely to be offered and undergo genetic counseling and testing (Ademuyiwa et al., 2020; Lau‐Min et al., 2023). Future well‐designed research both within and comparing diverse populations in racial/ethnic, gender, religion, age, education level, socioeconomic background, languages spoken, geographic location, disability identification, and other identities is needed to accurately reflect the populations to which guidelines may apply. Furthermore, the NSGC has also outlined organizational as well as short‐ and long‐term goals for improving diversity, equity, and inclusion within the field of genetic counseling (Channaoui et al., 2020). Ultimately, addressing the inequities among patient populations cannot be achieved in the absence of furthering cultural competency, inclusion, and diversity within the genetic counseling workforce.

### 6.2 “Common” versus “rare” hereditary cancer predisposition syndromes

The existing, high‐quality studies of genetic counseling outcomes for individuals at risk for hereditary cancer primarily include individuals at risk for common hereditary cancer syndromes, that is, hereditary breast and ovarian cancer. One challenge recognized by the workgroup was applying available evidence to the entire population of individuals at risk for hereditary cancer syndromes, including rare conditions and pediatric populations. For example, a recent systematic review for genetic testing in childhood cancer demonstrated the lack of significant additional harm and the presence of positive outcomes, while the authors also acknowledged the need for studies with larger sample sizes to assess the impact on testing across a diversity of childhood cancer predisposition syndromes (Van Hoyweghen et al., 2024).

### 6.3 Risk of bias

An NSGC‐endorsed guideline on the role of genetic counseling may have a risk of bias, with professionals having an interest in the strength and direction of the recommendation. Published protocols for future systematic evidence reviews, with continued guidance from the NSGC‐PGC, will allow for transparency and reproducibility. Incorporation of diverse patient, provider, and healthcare administrators in future workgroups, as well as the consideration of joint guidelines with other professional groups and national organizations, will allow for a more objective process.

## 7 FUTURE DIRECTIONS: “A CALL TO ACTION”

Research priorities for future studies were listed in Culver et al. (2024) and have been further detailed below. Of note, both the CHARM (Cancer Health Assessments Reaching Many) and BRIDGE (Broadening the Reach, Impact, and Delivery of Genetic Services) randomized‐controlled studies are currently in progress, which aim to address outcomes of varying service delivery models for cancer genetics services (Kaphingst et al., 2021; Mittendorf et al., 2021). Further, results of the MAGENTA (MAking GENetic Testing Accessible) study recently reported findings on the effectiveness of online genetics education compared with pre‐ and post‐test genetic counseling for participants unaffected with cancer undergoing genetic testing due to family history (Swisher et al., 2023). Findings of this study demonstrated non‐inferiority of the web‐based educational module as measured by anxiety, depression, or decisional regret at 3 months, as well as higher uptake of genetic testing in the groups that did not require genetic counseling. Limitations of MAGENTA include internet recruitment, leading to a highly educated White population, and the lack of ability to look at health services outcomes of genetic testing, such as ordering the correct test. The results of these and other well‐designed studies in the future will help to show the outcomes of these interventions and provide guidance for planning on how, when, and for whom genetic counseling for hereditary cancer may be most beneficial.

### 7.1 Guideline‐focused aims

- Establish evidence‐based guidelines related to hereditary cancer focusing on the care of individuals with specific cancer syndromes at different points in their lives to ensure comprehensive and appropriate recommendations pertaining to each specific condition. Different individuals with different syndromes may have different genetic counseling needs throughout their lifespans.

### 7.2 Research‐focused aims

- Separate outcomes associated with genetic counseling (pre‐test and/or post‐test) and genetic testing. Well‐designed studies (including randomized and/or observational case–control and cohort) to evaluate the patient‐ and health‐related outcomes associated with genetic testing alone versus a form of genetic counseling with genetic testing. This study design would be able to assess patient‐oriented outcomes. Comparator groups could include participants receiving genetic testing only; pre‐test genetic counseling with genetic testing; post‐test genetic counseling with genetic testing; pre‐ and post‐test genetic counseling and genetic testing; no genetic testing, no genetic counseling (at‐risk patients); pre‐test genetic counseling with no genetic testing. Of note, the MAGENTA study has studied the question related to non‐mandated pre‐test GC and post‐test GC only for participants with clinically negative results (Swisher et al., 2023).
- Assess the breadth of patient‐reported and health services‐related outcomes. For example, further research is needed to identify health services‐related outcomes, such as accurate risk assessment, correct tests ordered, and reduction of inappropriate services. Explorations could utilize electronic medical record review, claims analysis, and patient reports.
- Compare GC delivered by genetics‐trained versus non‐genetics‐trained providers. Well‐designed studies could compare outcomes associated with services provided by certified genetic counselors (CGC versus a non‐CGC) provider with or without genetics health training. The design could include outcomes associated with pre‐ and/or post‐test services. These studies could address patient‐, provider‐, and health services‐related outcomes.
- Expand research to include a more diverse, equitable, and inclusive lens, improve justice to historically excluded populations, and produce evidence representing diverse identities, perspectives, backgrounds, observances, and physical and lived experiences. Qualitative study designs can identify barriers which can be by well‐designed observational studies (case–control, cohort) within diverse populations to assess patient‐reported outcomes.

Real‐world constraints, including the acceptability of genetic counseling and specific payor policies, should be considered in the feasibility of large, clinically derived patient populations. Well‐designed, single‐arm observational studies with pre‐ and post‐test designs, as well as observational comparative studies with consistent use of validated measures for outcomes, would assist in future meta‐analyses. Examples of previously used outcome measures include the State Trait Anxiety Inventory (STAI), Decisional Conflict Scale, Perceived Personal Control Scale, Brief Symptom Inventory (BSI), Center for Epidemiologic Studies Depression Scale (CES‐D), 12‐item General Health Questionnaire (GHQ‐12), Hospital Anxiety and Depression Scale (HADS), Cancer Worry Scale, Impact of Event Scale, Genetic Counseling Outcome Scale (GCOS), and others (Athens et al., 2017; Culver et al., 2024; Madlensky et al., 2017; McAllister et al., 2011). Electronic medical record review and claims analyses review could provide outcomes related to health behavior. Validation in diverse populations would be essential for the recommended research in non‐White, non‐female participant groups. Every aspect of research design should include patient considerations and perspectives.

## 8 CONCLUSIONS

An NSGC‐sponsored effort was undertaken to develop an evidence‐based guideline relevant to the outcomes of genetic counseling for individuals at risk for developing hereditary cancer. As part of this process, a systematic evidence review and meta‐analysis were performed, which revealed gaps in the evidence for critically ranked outcomes of genetic counseling. As such, a GRADE method guideline related to the process of genetic counseling for individuals at risk for hereditary cancer was not completed. Rather, the workgroup has outlined the issues, insights, and recommendations from this process. Study designs to address the benefits, risks, and limitations of genetic counseling targeting the patient‐, provider‐, and system‐related outcomes are provided. By describing the process and highlighting the types of well‐designed studies from which to develop guidelines, the workgroup's intention is to spur future projects to address the identified gaps in the literature as a basis for future guideline‐related efforts.

## AUTHOR CONTRIBUTIONS

B. Levin and K. Wolfe Schneider agree to be accountable for all aspects of the work in ensuring that questions related to the accuracy or integrity of any part of the work are appropriately investigated and resolved. B. Levin, E. Salo‐Mullen, J.O. Culver, R.N. Kurz, P. Brock, R. Demsky, S. Lloyd, G. Lopez, P. L. Mai, and K. Wolfe Schneider provided substantial contributions to the conception and design of the work; drafting and revising it critically for important intellectual content; and final approval of the version to be published.

## CONFLICT OF INTEREST STATEMENT

The author group composition is in compliance with the National Society of Genetic Counselors Practice Guidelines Committee Conflict of Interest Policy. This policy requires all proposed authors to disclose conflicts of interest prior to selection and imposes thresholds for conflicts of interest with the potential for direct, personal financial benefit, or other real or perceived conflicts of interest, through the development of the document. B. Levin, E. Salo‐Mullen, J.O. Culver, R.N. Kurz, P. Brock, R. Demsky, S. Lloyd, G. Lopez, P. L. Mai, and K. Wolfe Schneider declare that they have no conflicts of interest.

## ETHICS STATEMENT

Human studies and Informed consent: No human studies were carried out by the authors for this article.

## Data Availability

Data sharing is not applicable to this article as no new data were created or analyzed in this study.
